# Nuclearization of β-catenin in ectodermal precursors confers organizer-like ability to induce endomesoderm and pattern a pluteus larva

**DOI:** 10.1186/2041-9139-4-31

**Published:** 2013-11-04

**Authors:** Christine A Byrum, Athula H Wikramanayake

**Affiliations:** 1 Department of Biology, College of Charleston, 58 Coming Street, Room 214, Charleston, SC 29401, USA; 2 Department of Biology, The University of Miami, 1301 Memorial Drive, Coral Gables, FL 33146, USA; 3 Department of Biology, 2538 The Mall, University of Hawaii at Manoa, Honolulu, HI 96822, USA

**Keywords:** β-catenin, Organizing center, Sea urchin, *Lytechinus variegatus*, Animal-vegetal axis, Development, Wnt signaling, Anterior-posterior polarity, Endomesoderm

## Abstract

**Background:**

In many bilaterians, asymmetric activation of canonical Wnt (cWnt) signaling at the posterior pole is critical for anterior-posterior (AP) body axis formation. In 16-cell stage sea urchins, nuclearization of β-catenin in micromeres activates a gene regulatory network that defines body axes and induces endomesoderm. Transplanting micromeres to the animal pole of a host embryo induces ectopic endomesoderm in the mesomeres (ectoderm precursors) whereas inhibiting cWnt signaling blocks their endomesoderm-inducing activity and the micromeres become ectoderm-like. We have tested whether ectopic activation of cWnt signaling in mesomeres is sufficient to impart the cells with organizer-like abilities, allowing them to pattern normal embryonic body axes when recombined with a field of mesomeres.

**Results:**

Fertilized eggs were microinjected with constitutively active *Xenopus β-catenin (actβ-cat)* mRNA and allowed to develop until the 16-cell stage. Two mesomeres from injected embryos were then recombined with isolated animal halves (AH) from uninjected 16-cell stage embryos. Control chimeras produced animalized phenotypes (hollow balls of ectoderm) and rarely formed skeletogenic mesoderm (SM)-derived spicules, endoderm or pigment cells, a type of non-skeletogenic mesoderm (NSM). In contrast, over half of the 0.5 pg/pL actβ-cat mesomere/AH chimeras formed a partial or complete gut (exhibiting AP polarity), contained mesenchyme-like cells similar to SM, and produced pigment cells. At three days, chimeras formed plutei with normal embryonic body axes. When fates of the *actβ-cat* mRNA-injected mesomeres were tracked, we found that injected mesomeres formed mesenchyme-like and pigment cells, but endoderm was induced. Higher concentrations of *actβ-cat* mRNA were less likely to induce endoderm or pigment cells, but had similar mesenchyme-like cell production to 0.5 pg/pL *actβ-cat* mesomere/AH chimeras.

**Conclusions:**

Our results show that nuclear β-catenin is sufficient to endow naïve cells with the ability to act as an organizing center and that β-catenin has both cell-autonomous and non-autonomous effects on cell fate specification in a concentration-dependent manner. These results are consistent with the hypothesis that a shift in the site of early cWnt signaling in cleaving embryos could have modified polarity of the main body axes during metazoan evolution.

## Background

**Figure 1:**
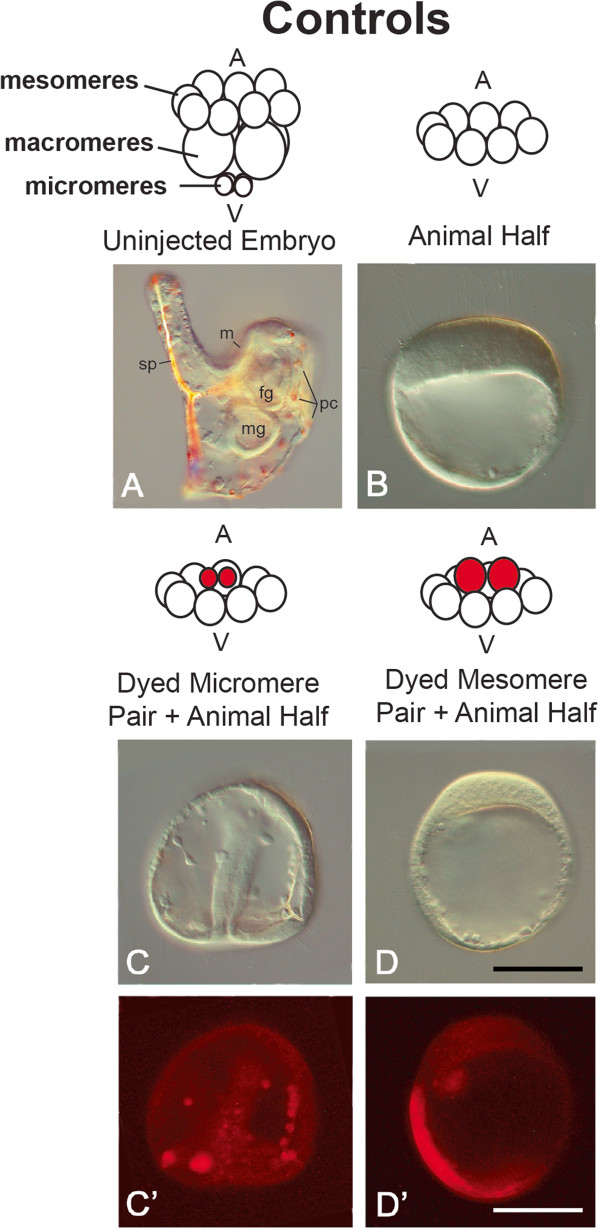
**Summary of the controls used in experiments. (A-D)** Controls, bright field images. **(C’, D’)** Controls, fluorescent images. **(A)** Uninjected embryo at 27 hours post fertilization (hpf). **(B)** The uninjected animal half (AH) forms a hollow ciliated ball of ectoderm lacking endomesoderm and patterning along the oral-aboral axis (dauerblastula), 24 hpf. **(C, C’)** A chimera was made at the 16-cell stage in which micromeres removed from an embryo injected with rhodamine-dextran (RDX) were recombined with the uninjected AH. This produces a nearly normal prism stage embryo, although few pigment cells were present in many individuals. The RDX-labeled micromeres contributed to mesenchyme-like cells and occasionally to pigment cells (not shown). **(D, D’)** When RDX-labeled mesomeres from a 16-cell embryo were recombined with an AH, the chimera formed a dauerblastula and most of the labeled cells remained in the ectoderm. Note the expanded apical plate at the animal pole **(B and D, D’)**. This is a typical feature of the dauerblastula. Orientations of the embryos are as follows: **(A)** Lateral view, oral side up. **(B, C, C’, D, D’)** All other images are frontal views with the animal pole oriented towards the top. Scale bars = 100 μm.

During metazoan embryogenesis establishing polarity early in development is critical for proper formation of the body plan. Abnormal placement or loss of these cues can have severe consequences such as disruption of normal cleavage patterns, cell-fate specification, and morphogenesis. In the sea urchin embryo two main body axes are established during early development: the anterior-posterior or AP axis and the dorsoventral or DV axis (also known as the oral-aboral axis). Formation of the AP axis is strongly influenced by the maternally specified animal-vegetal (AV) axis of the egg [1,2]. The distribution of critical maternal determinants required for AP axis patterning of the sea urchin embryo was revealed in classical embryological experiments where the unfertilized egg was bisected equatorially, and each half was fertilized. In these experiments, the vegetal halves produced embryos that were almost normal, but the animal halves (AHs) developed into hollow ciliated balls of ectoderm (a dauerblastula) lacking endomesoderm and ectoderm-derived structures such as the stomodeum and ciliary band [2,3], thus, demonstrating that cues for global patterning of the embryo exist in vegetal portions of the unfertilized egg. Although AP polarity is established early, the presence of this axis is not morphologically evident until the 16-cell stage. Each of the first three cleavages produce equal size daughter blastomeres, but at the fourth cell division, unequal cleavage of animal and vegetal blastomeres produces an embryo with different-sized cells arranged into three tiers. Eight medium-sized cells called mesomeres form a ring at the animal pole and lie above four large cells called the macromeres. At the 16-cell stage, four small cells called micromeres (Figure 1) are born at the vegetal pole and signaling by these cells initiates patterning along the AP axis. Cell-fate specification in the three tiers is stereotyped at the 16-cell stage. The mesomere progeny will give rise to ectodermal derivatives, whereas micromere and macromere progeny form the endomesoderm. By the late blastula stage, cell-cell signaling establishes distinct territories arranged as follows from the animal to vegetal pole: anterior neuroectoderm (ANE) and posterior neuroectoderm (PNE) both arise from the mesomeres; a small portion of the ectoderm, most of the endoderm, and non-skeletogenic mesoderm (NSM) form from the macromeres; and skeletogenic mesoderm (SM) and germline cells originate from the micromeres [4-10].

Although the sea urchin embryo establishes AP polarity quite early, the DV axis is not specified until early cleavage, and unlike the AP axis, specification of the DV axis remains labile until the 16-cell stage [11,12]. At the gastrula stage, presence of the DV axis first becomes evident when the embryo develops bilateral symmetry and the oral ectoderm (ventral surface) starts to flatten [13]. By the pluteus stage, the structure of the ectoderm is modified, producing two main ectodermal territories: the oral ectoderm (ventral) and aboral ectoderm (dorsal). Cells of the oral ectoderm produce the stomodeum, whereas the aboral ectoderm differentiates into a squamous epithelium. In areas where the two territories meet, a third form of ectoderm arises, ciliated band ectoderm [8,14]. Finally, a fourth ectodermal territory arises when the ANE differentiates to produce numerous serotonergic neurons and the apical tuft of the neural plate [8,15,16].

A key signal transduction pathway influencing embryonic polarity in metazoans is the canonical Wnt (cWnt) pathway [8,17-23]. This pathway is normally activated when a Wnt ligand binds to the cell surface co-receptors Frizzled (Fz) and Arrow/LRP 5/6 to transduce a signal that ultimately inhibits the ability of glycogen synthase kinase-3β (GSK-3β) to phosphorylate and target β-catenin for degradation by the proteosome pathway. With the loss of GSK-3β activity, β-catenin becomes stable in the cytoplasm and then moves into the nucleus where it interacts with the transcription factor TCF/LEF to activate target gene expression. During sea urchin embryogenesis, the location of nuclear β-catenin changes as the embryo develops, but it is consistently restricted to cells that will form endoderm or mesoderm. Nuclear β-catenin is first detected in the micromeres at the 16-cell stage, and by the 32-cell stage it has accumulated in nuclei of the macromeres as well. In 60-cell embryos, equatorial division of the macromeres produces the veg1 (anterior) and veg2 (posterior) cells. Less accumulation of nuclear β-catenin is found in the veg1 cells and following the seventh cleavage, cWnt signaling is downregulated in the veg1 progeny. As embryogenesis continues, the pattern changes again, and by the end of primary invagination, nuclear β-catenin is restricted to a ring of veg1 cells surrounding the blastopore [24].

Signaling through the cWnt pathway is necessary for both AP and DV patterning in the sea urchin embryo. If nuclear localization of β-catenin is blocked by the overexpression of C-cadherin, AP patterning is lost and embryos are animalized [24,25]. In this case, endoderm and mesoderm are both absent and the embryo consists only of ectoderm. Cuboidal epithelial cells are present at one end, but most cells resemble the ANE and produce serotonergic neurons [15]. In addition DV polarity is disrupted due to ectopic expression of regulatory factors involved in ANE specification [10,15,26,27]. With loss of cWnt signaling, the ANE regulatory factor FoxQ2 (an ANE regulatory factor) is no longer downregulated and the expression domain of this transcription factor expands such that it overlaps with and disrupts expression of Nodal (the DV axis determinant) [26].

In sea urchin embryos, cWnt signaling is also critical for patterning of the AV axis. For example, if actβ-cat (a stabilized form of β-catenin that cannot be negatively regulated by GSK-3β) is overexpressed in whole embryos, they become vegetalized, produce high amounts of endomesoderm at the expense of ectoderm [25], and are radialized (body axis formation is disrupted and serotonergic neurons are lost, likely due to loss of the ANE) [8,15]. The normal phenotype can be rescued simply by co-injecting the fertilized egg with *actβ-catenin* and *C-cadherin* mRNA. Both polarity and normal endomesoderm formation are recovered [25]. Thus, cWnt signaling is clearly necessary for proper patterning of the axes and specification of the endomesoderm. In similar experiments, overexpressing actβ-cat in an isolated AH induces ectopic endoderm and mesoderm, indicating that activation of the cWnt pathway is sufficient for endomesoderm formation [25]. Similarly, treating embryos, AHs, or isolated mesomere pairs with lithium chloride (lithium), a chemical that activates cWnt signaling, causes ectopic expression of endoderm and mesoderm [11,28-30]. Manipulating other key components of the cWnt pathway such as Dishevelled [31,32], Wnt6 [33], GSK-3β [34], Lef/TCF [35,36], Axin [10], and Fz [37] have all produced results consistent with the cWnt pathway playing a key role in endomesoderm specification and in regulating pattern formation along the AP axis in the sea urchin embryo.

Investigators have also examined roles of the cWnt pathway in promoting signaling between the micromeres and neighboring cells. The ability of micromeres to induce formation of endomesoderm in neighboring cells is well-established [38-41] and is unique to the micromeres unless the embryo is perturbed. When micromeres are transplanted to the animal pole of a normal 8- to 32-cell-stage embryo, a second fully differentiated archenteron is induced from the mesomeres, and the transplanted micromeres will cell-autonomously differentiate secondary skeletal structures that are positioned correctly relative to the ectopic archenteron. To determine if nuclear β-catenin is required for these properties of the micromeres Logan *et al*. [24] overexpressed C-cadherin by mRNA injection into a fertilized egg to block cWnt signaling and then transferred micromeres from this embryo to the animal pole of an uninjected 8-cell embryo. Although transplantation of normal micromeres induced endomesoderm formation, transplantation of micromeres expressing C-cadherin failed to induce formation of an ectopic gut. The injected micromeres also failed to ingress or form skeletogenic cells and contributed to the epidermal layer at the animal pole. Based on this, Logan *et al*. concluded that cWnt signaling is necessary for the ability of micromeres to signal neighboring cells and for these cells to differentiate into SM cells. In sum, these results indicate that nuclear β-catenin is required for the organizer-like activity of the micromeres, and moreover, that in the absence of cWnt signaling, these cells adopt an ectodermal cell fate.

In this study, we test whether selective nuclear localization of β-catenin is sufficient to instill mesomeres with micromere-like signaling abilities, capable of patterning the AP and DV axes. We found that mesomeres expressing actβ-cat can induce formation of endoderm and some NSM cells, and moreover, that these cells give rise to SM cells. Acting like an organizing center the actβ-cat-expressing mesomeres established proper AP and DV polarity in an embryo derived from mesomeres. This work further establishes the crucial importance of cWnt signaling in sea urchin development and demonstrates that ectopic nuclear accumulation of β-catenin is sufficient to endow mesomeres with many of the capacities typically seen only in the micromeres, the organizing center of the embryo during early AV axis patterning.

## Methods

### Care of animals/embryos

*Lytechinus variegatus* were imported from Duke Marine Lab (Beaufort, NC, USA) or the Florida Keys, FL, USA (KP Aquatics) and maintained in aquaria at room temperature. To induce spawning, 1 mL of 0.5 M potassium chloride was injected into the adult urchin up to four times. Eggs were collected in artificial seawater (ASW) and maintained at room temperature. Sperm were collected dry and stored on ice or at 4°C; 50 μL of sperm diluted 1:1000 in ASW was added to approximately 10 mL of ASW containing eggs. Embryos were raised in an incubator at 22°C.

### Constructs and microinjection

The plasmid containing cDNA of an activated form of *Xenopus* β-catenin (actβ-cat) was a gift of D Kimelman [42]. This construct is a stabilized form of β-catenin in which four key serine and threonine residues at the amino terminus have been mutated to prevent phosphorylation and ubiquitination [42], thus, this form of β-catenin cannot be degraded and will accumulate in the nuclei of cells when overexpressed [31]. Plasmids containing this construct were linearized and mRNA was transcribed using mMessage mMachine kits (Ambion, Austin, TX, USA). The mRNA was isolated by phenol-chloroform extraction and quick-spin column purification (Roche, Indianapolis, IN, USA) followed by isopropanol precipitation. Prior to injection, mRNA was suspended in 25 to 40% glycerol in RNase-free water. To track the injected mRNA, 2 pg/pL of rhodamine-labeled dextran (RDX) was also added to this solution. *Actβ-cat* mRNA was injected at a concentration of 0.5 or 1.0 pg/pL.

Embryos were prepared for microinjection as follows. Prior to fertilization, eggs were washed three times in ASW and then treated with low-pH seawater (5.0) to remove the jelly coat as described [43]. Eggs were then transferred to a 1% protamine sulfate-coated dish and fertilized in ASW containing 0.5 mM 3-amino 1,2,4-triazole to prevent hardening of the fertilization envelope. Fertilized eggs were immediately pressure injected with *actβ-cat* mRNA equal to approximately 1 to 2% total embryo volume.

### Microsurgery

**Figure 2:**
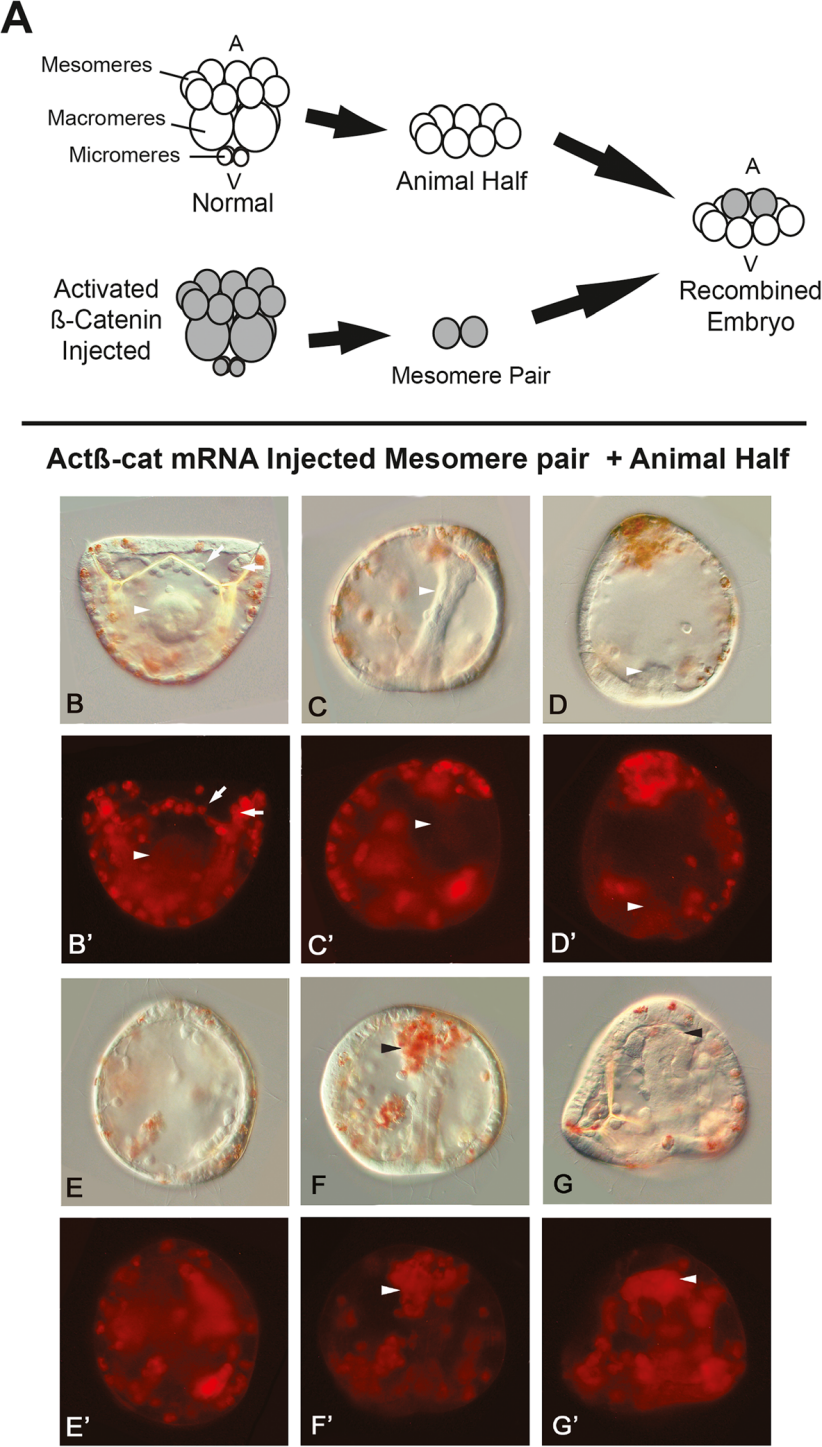
**Summary of cell transplantation experiments and results at 23 to 27 hours post fertilization. (A)** A mesomere pair from a 16-cell embryo injected with *actβ-cat* mRNA (shaded embryo) was recombined with the eight mesomeres of an uninjected animal half (AH). **(B-G)** Bright field images of chimeras in which a labeled mesomere pair injected with 0.5 pg/pL *actβ-cat* mRNA was recombined with an uninjected AH. **(B’-G’)** Corresponding fluorescent images. Labeled/injected mesomeres formed mesenchyme-like cells and pigment cells. Absence of label in the gut (gut indicated with arrowheads in **B’, C’, D’**) suggests that this tissue is induced in mesomeres from the uninjected AH. Some embryos were similar to uninjected controls **(B, B’, C, C’, F, F’, G, G’)**, forming a complete gut, mesenchyme-like cells (indicated with arrows in **B’**) that produced spicules, and pigment cells (development in the injected cells and transplants is delayed compared to that in uninjected embryos). Others **(D, D’)** only formed a partial gut or lacked a gut **(E, E’)**. **(F, F’-G, G’)** In some, the labeled cells were observed at the tip of the archenteron (arrowheads in **F’ and G’**). In this Figure, labeled mesenchymal cells are also clearly evident in the blastocoel. These are likely skeletogenic mesoderm and/or non-skeletogenic mesoderm cells such as pigment cells, but it is interesting that, unlike other chimeras, many of the labeled cells in these individuals remain concentrated at the tip of the archenteron. Orientation: **(B, B’)** vegetal view, **(C-E, C’-E’)** lateral views. **(F-G, F’-G’)** Frontal views with the animal pole at the top of the photograph.

All micro dissections were performed in agar-coated dishes using a *knife* made of hair mounted on a wooden applicator stick. Embryos at the 8-cell stage were placed in hyaline extraction media [44] for two minutes to weaken the hyaline layer, and then transferred into calcium-free seawater [44]. At the 16-cell stage, a pair of mesomeres were removed from an *actβ-cat* mRNA-injected embryo and the AH (8 mesomeres) was isolated from an uninjected 16-cell embryo. The *actβ-cat* mRNA injected mesomeres were then placed onto the uninjected AH and left undisturbed until they firmly adhered (Figure 2A). Afterwards the chimera was transferred from calcium-free seawater to ASW. Embryos were visually assessed for the presence of endoderm, mesenchyme-like cells/spicules, pigment cells, and the ability to swim at the prism stage using a Zeiss Axiovert 200 inverted microscope, or in some of the later experiments, at 3 days. Controls were produced in a similar manner in which uninjected cells or dye-injected cells were transplanted to uninjected AHs.

### Statistical analysis

The statistical analyses that appear in Table 1 and in the text (where frequency of phenotypes in 0.5 pg/pL *actβ-cat* mRNA-injected chimeras was compared to that in 1 pg/pL *actβ-cat* mRNA-injected chimeras) were performed using a Chi-square goodness-of-fit test. In Table 1 all stages were compared to untreated AHs. *P*-values <0.05 were considered significant.

**Table 1:** **Cell transplantation data for 23 to 27 hours post fertilization *Lytechinus variegatus *** **embryos**

**Embryo type**	**Animalized**	**Complete or partial gut**	**Mes**-**like cells**	Spicules^***^	**Pigment cells**	**Swimming**
Uninjected AH	92% (33/36)	8% (3/36)	11% (4/36)	0% (0/25)	6% (2/36)	14% (5/36)
Uninjected micro pair + uninjected AH	0% (0/36)^**^	63% (10/16)^**^	100% (16/16)^**^	100% (16/16)^**^	25% (4/16)^**^	91% (10/11)^**^
Uninjected meso pair + uninjected AH	79% (11/14) (3 of these had a few loose cells)	14% (2/14)	29% (4/14)^*^ 7% (1/14) solid	0% (0/11)	0% (0/14)	7% (1/14)
0.5 *Actβ*-cat meso pair + uninjected AH	18% (9/50)^**^	54% (27/50)^**^	82% (41/50)^**^	82% (41/50)^**^	78% (39/50)^**^	82% (41/50)^**^
1.0 *Actβ*-cat meso pair + uninjected AH	17% (4/23)^**^	30% (7/23)^**^	83% (19/23)^**^	59% (13/22)^**^	52% (12/33)^**^	70% (16/23)^**^

Mes-like cells indicated the presence of individual cells in the blastocoel. ^*^Chi-square test indicates significant difference of 0.05>P>0.01 when compared to uninjected animal half (AH). ^**^Chi-square test indicates significant difference of 0.01>P>0.001 when compared to the uninjected AH. ^***^Because the expected value was 0, this produces a numerator of 0 in the Chi-square test, so a larger expected value of 1 was substituted to perform the test for significance.

## Results

### Mesomere descendents expressing nuclear β-catenin are micromere-like, but also form NSM cells

At the 16-cell stage, cWnt signaling is activated at the vegetal pole in the sea urchin embryo when β-catenin translocates from the cytoplasm to the nucleus in the micromeres [24]. When AHs are produced from embryos at this stage and these mesomeres are cultured in isolation, they develop into the classic dauerblastula phenotype and rarely form any endomesoderm when assayed at 23 to 27 hours post fertilization (hpf), whereas undisturbed control embryos have reached the pluteus larval stage at this time (Figure 1A, B). When AH controls were generated, we found that most formed dauerblastulae (92%, n = 33/36) (Table 1, Figure 1B) and only 8% (n = 3/36) formed partial or complete guts, 6% (n = 2/36) produced pigment cells, 11% (n = 4/36) formed mesenchyme-like cells, and 0% (n = 0/25) produced spicules (Table 1). These AHs failed to polarize the oral-aboral axis and did not form a stomodeum or ciliary band (Figure 1B). In this study, formation of a partial or complete gut was based on visual evaluation of the embryos. When cells were invaginating and/or forming an archenteron but had not completed gastrulation, the embryo was scored as having a partial gut. If the archenteron extended across the entire blastocoel, the embryo was scored as having a complete gut. While it is possible that in some of the cases in our studies we could have missed cells that were specified as endomesoderm but had not undergone invagination, the presence or absence of an archenteron in *L. variegatus* is unequivocal due to the optical clarity of the embryos. The presence of mesenchyme-like cells was recorded if individual mesenchyme-like cells had accumulated in the blastocoel. It is noteworthy in some instances that a portion of these cells may also form from the NSM. For this reason, we have also evaluated the presence or absence of spicules and these data are shown in Table 1. Based on the work of others [15,26,27,45] (reviewed in [8]) it is clear that signals to induce patterning along the oral-aboral or dorsoventral axis (such as formation of the stomodeum and the ciliary band) are regulated by cWnt-dependent signals.

Previous studies have also shown that if 16-cell-stage micromeres are transplanted to the AH of an intact embryo [38,39] or if these cells are recombined with an isolated AH [38,41,46], the transplanted micromeres induce endomesoderm formation in the mesomeres. When we transplanted a pair of normal micromeres onto an AH (micromeres (2)/AH) (n = 16), none of the chimeras formed dauerblastulae, and in 63% (n = 10/16) of the cases, a partial or complete gut was formed. All of these chimeras produced mesenchyme-like cells (n = 16/16) and spicules (n = 16/16) (Table 1, Figures 1C and 1C’). Pigment cells were less common in the chimeras compared to normal embryos and only occurred in 25% (n = 4/16) of the cases. When micromere pairs were labeled with RDX, the label typically ended up in mesenchyme-like cells. In 71% (n = 5/7) of these cases where a partial or complete gut formed, the label was also present in a portion of the archenteron tip (data not shown). Failure of the micromeres to activate endoderm or pigment cell formation may be caused by loss of a transplanted micromere or it may be that more micromeres (three or four) are needed to fully activate endomesoderm formation in *L. variegatus* AHs.

In sharp contrast to the dramatic morphological changes elicited by micromeres transplanted onto an AH, when a pair of mesomeres was combined with an intact AH (n = 14), a majority of these chimeric embryoids formed dauerblastulae (79%, n = 11/14) and 7% (n = 1/14) were solid (the outer cell layer had two areas with longer cilia but fairly uniform cell thickness; the center of the chimera was occluded with a homogeneous mass of cells) (Table 1, Figures 1D and 1D’). Three of the dauerblastulae contained one to three loose cells. These were categorized as dauerblastulae despite the presence of the few cells, based on the fact that they each exhibited an expanded apical plate, the loose cells did not appear to be mesenchyme-like, and these individuals lacked endodermal or mesodermal structures such as the gut, pigment cells or spicules. Another chimera was solid (7%, n = 1/14) and is included as a separate category in Table 1. In this individual the blastocoel was completely occluded. These cells were not likely to be SM or NSM cells as this embryo failed to form spicules or pigment cells. Instead, we suspect that this conformation indicated an embryo either damaged by exposure to the hyaline extraction medium or damaged during surgery. Mesenchyme-like cells were present in 29% (n = 4/14) of these chimeras, however none (n = 0/11) produced spicules. Endoderm was only observed in 14% of the embryos (n = 2/14) (Table 1). Although only one region of the ectoderm is thicker in a typical dauerblastula, some of these controls had two thickened areas in the ectoderm. In four of these chimeras, RDX-labeled mesomere pairs were recombined with unlabeled AHs. Labeled mesomere pairs showed that in one case, the transplanted mesomeres contributed to the single thickened ectodermal region in the embryo. In another case labeled mesomeres were restricted to one of two thickened regions in the ectoderm. In a third case, dye was in the thin ectodermal region of the dauerblastula, and in the final case, the embryo was solid and the transplanted mesomere progeny formed the central cell mass as well as a portion of the ectoderm. None of the labeled chimeras formed endoderm.

Logan *et al*. [24] demonstrated that blocking nuclear β-catenin in micromeres of the sea urchin *L. variegatus* led to the loss of the endomesoderm-inducing activity of these cells, and moreover, converted these cells into an ectoderm-like morphology. To determine whether selective nuclear localization of β-catenin could transform a pair of mesomeres into cells with micromere-like activity, chimeras were first generated by recombining two mesomeres from an embryo injected with 0.5 pg/pL *actβ-cat* mRNA with an uninjected AH (0.5 pg/pL *actβ-cat* mesomere/AH) (n=50) (Figure 2A-G and B’-G’, Table 1). By 23 to 27 hpf, these embryos had the following characteristics: 54% (n = 27/50) formed partial or complete guts, 82% (n = 41/50) contained mesenchyme-like cells, 82% (n = 41/50) formed spicules, 78% (n = 39/50) produced pigment cells, and only 18% (n = 9/50) were animalized. In a subset of the 0.5 pg/pL *actβ-cat* mesomere/AH embryos (n = 11) the transplanted mesomere pair was co-injected with 2 pg/pL RDX. At 23 to 27 hpf, all of the *actβ-cat* mRNA-injected mesomere pairs became mesenchyme-like cells and pigment cells (Figure 2B’-G’). In five of the eleven cases, embryos formed complete guts, but although the label was sometimes present in the NSM-like cells forming at the tip of the archenteron, it was not seen in other areas of the foregut, mid-gut, or hindgut (Figure 2F’-G’).

**Figure 3:**
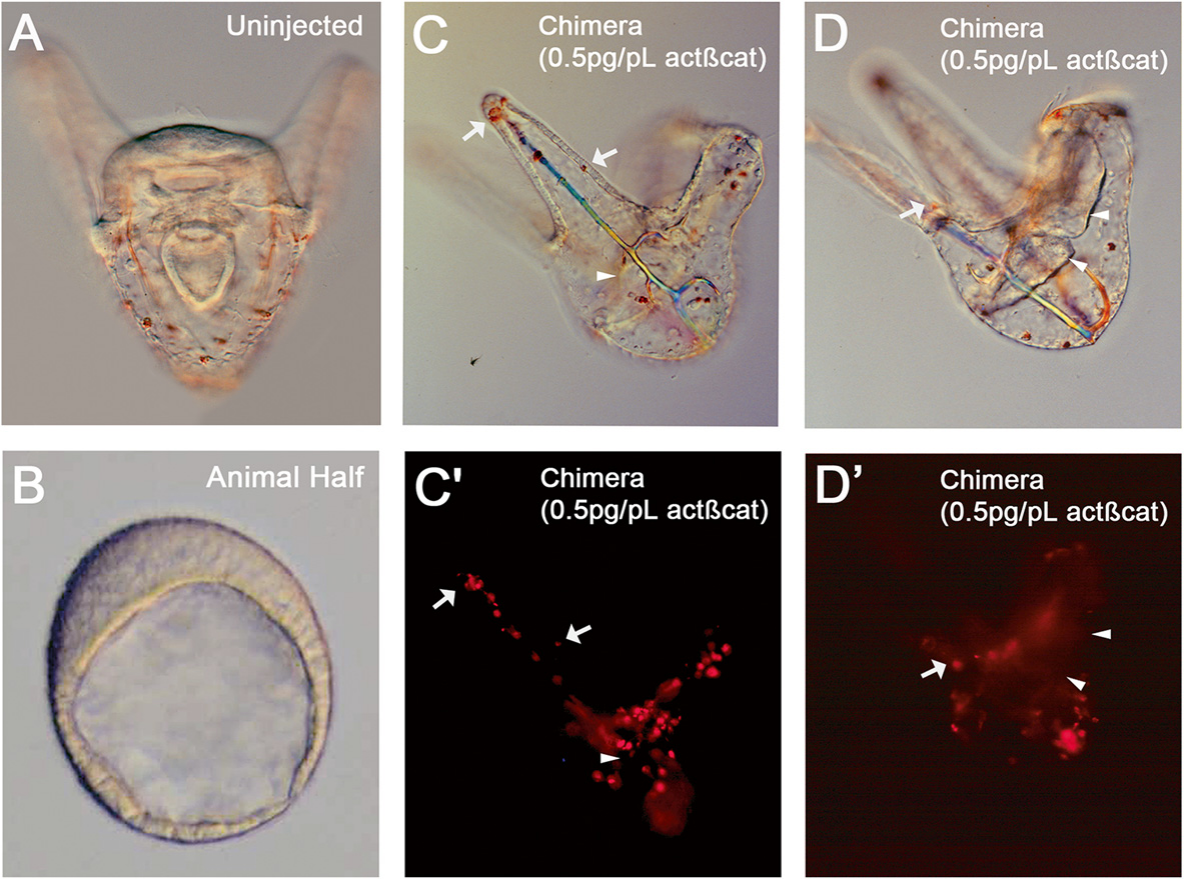
**Results of cell transplantation experiments at 3 days post fertilization. (A)** Uninjected control. **(B)** Animal half (AH) control. **(C)** Bright field image of a 3-day chimera formed from a labeled mesomere pair injected with 0.5 pg/pL *actβ-cat* mRNA recombined with an uninjected AH. Note that features such the spicules, a tripartite gut, and pigment cells are all present. Larvae appear to be normal. **(C’)** Fluorescent image shows that the *actβ-cat* mRNA-injected mesomeres gave rise to mesenchyme-like cells and pigment cells (arrows in **C, C’, D, and D’** indicate pigment cells). **(D, D’)** Different focal plane in the same embryo, showing that cells in the gut are not derived from the *actβ-cat* mRNA-injected mesomeres. Arrowheads in **C, C’, D, and D’** indicate the induced, unlabeled gut.

To determine whether characteristics of the RDX plus 0.5 pg/pL *actβ-cat* mRNA-injected mesomere/AH embryos changed during later developmental stages, five additional recombinations were performed and these chimeras were allowed to develop for three days. The 3-day chimeric embryos were smaller, but looked morphologically similar to uninjected control plutei (Figure 3). Again, the labeled *actβ-cat* mRNA-injected mesomere pairs produced progeny that formed mesenchyme-like cells and pigment cells. Labeled cells were not found in the coelomic pouches, endoderm, or muscles (Figure 3D, D’). This observation is notable because it supports the conclusion that addition of an *actβ-cat* mRNA-injected mesomere pair is sufficient to allow what appears to be proper specification of the embryonic body plan. These *act-βcat* mRNA-injected mesomeres act like an organizing center in the chimera, rescuing not only specification of the endomesodermal structures, but also restoring formation of the AP and DV axes.

Although micromeres combined with an AH develop mesenchyme-like cells (Figure 1C, C’, Table 1), *actβ-cat* mRNA-injected mesomeres combined with the AH become at least two cell types, forming both mesenchyme-like cells that appear to be SM and pigment cells, a subpopulation of the NSM (Figures 2 and 3). As mentioned above, in the *actβ-cat* mesomere/AH chimeras, two of the cell types typically derived from the NSM, the coeloms and esophageal muscle, do not form from the *actβ-cat* mRNA-injected mesomere pair (Figures 3D, D’). It is interesting that in our chimeras the NSM is partitioned so that subpopulations derived from the injected mesomeres (which experienced cWnt signaling) became pigment cells whereas the other NSM cell types were induced and derived from cells that did not receive the early cWnt signal.

### Induction of the gut is reduced in chimeras with increased nuclear β-catenin

**Figure 4:**
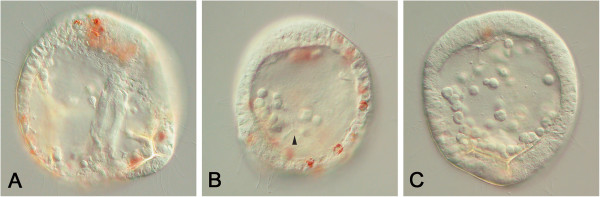
**Chimeras generated using higher levels of *actβ-cat *(1.0 pg/pL). (A)** Some embryos appeared to be nearly normal, forming spicules, a gut, and pigment cells. **(B)** In other cases, the gut was absent, but many pigment cells and some skeletal elements (arrowhead) are present, or **(C)** few pigment cells formed. All images are lateral views.

Previous studies had shown a concentration-dependent effect of β-catenin on patterning mesomeres [25]. Hence, to determine if mesomeres expressing different concentrations of β-catenin had distinct signaling activities, we produced mesomere pair + AH-chimeras (n = 23) using higher (1.0 pg/pL) concentrations of *actβ-cat* mRNA (Figure 4A-C). Chi-square analysis indicated that the chimeras produced with higher concentrations of *actβ-cat* mRNA (1.0 pg/pL) did not differ significantly from the 0.5 pg/pL *actβ-cat*-chimeras in number of mesenchyme-like cells present, tendency to produce animalized embryoids, or in ability to swim (Table 1). Significant differences were observed between the two groups in spicule formation (*P* <0.05), production of a complete or partial gut (*P* <0.01), and production of pigment cells (*P* <0.01). Spicule formation was observed in 59% (n = 13/22) of the 1.0 pg/pL *actβ-cat* chimeras, whereas this occurred in 82% (n = 41/50) of the 0.5 pg/pL *actβ-cat* mRNA chimeras. Also, higher concentration chimeras were less likely to form a complete or partial gut (30% or n = 7/23), whereas 54% (n = 27/50) of the 0.5 pg/pL-embryos formed a complete or partial gut. Finally, pigment cells were only observed in 52% of the cases (n = 12/23) whereas they were observed more often in 0.5 pg/pL *actβ-cat* mRNA chimeras (78% or n = 39/50). In images of these embryos, an average of 8.62 (± 6.75 SD) pigment cells were visible in the 1.0 pg/pL *actβ-cat* chimeras (n = 13 cases evaluated). There was quite a bit of variation in this character as is evident from the high SD. In 6/13 of these chimeras the number of pigment cells ranged from 0 to 5 and, in 5/13 chimeras, the number of pigment cells ranged from 14 to 19. This was far less than had been found in the 0.5 pg/pL-*actβ-cat* chimeras (29.23 ± 10.82 SD, n = 22 cases), but much higher than the numbers observed in the micromere/AH chimeras (0.13 ± 0.35 SD, n = 15 cases), or the uninjected mesomere/AH chimeras (0.0, n = 7 cases). In the 1.0 pg/pL-*actβ-cat* chimeras, those with higher numbers of pigment cells often had a more fully formed gut, but there were also cases where the number of pigment cells was high and no gut was observed. Thus, chimeras in which the transplanted mesomere was injected with higher levels of *actβ-cat* mRNA were similar in many ways to those injected with 0.5 pg/pL *actβ-cat* mRNA, but at the higher *actβ-cat* concentration endoderm formation was less frequent and pigment cell formation was not as common.

Note that the frequency of animalized phenotypes is similar at both concentrations, occurring in 17 to 18% of the cases. This likely reflects the failure rate of the recombination procedure. When chimeras were co-injected with *actβ-cat* mRNA and RDX, the label was often absent in the animalized embryos, suggesting that the labeled mesomere or micromere pair failed to adhere with the AH and fell off during culture.

## Discussion

### Ectopic activation of cWnt signaling in mesomeres imparts these cells with micromere-like activity

A role for cWnt signaling in endomesoderm specification during embryogenesis was first demonstrated in the sea urchin [24,25] and reviewed in [47]. These early investigations showed that loss of cWnt signaling produced the classical animalized phenotype where the nuclear β-catenin-depleted embryos did not express markers of endomesoderm or aboral ectoderm. Analysis of these embryos also showed that they failed to form stomodea and ciliary bands, indicating that cWnt signaling is necessary for normal development of both the AP and the DV axes. In more recent studies, sensitive lineage-specific molecular markers were used to analyze embryos animalized by loss of cWnt signaling and these experiments confirmed disruption of the embryonic lineages as described above. Intriguingly, molecular analysis of cWnt-disrupted embryos also detected an expansion of the ANE territory with most cells in these embryos expressing neuroectodermal markers [15]. These studies indicated that cWnt signaling was critical for early patterning in the sea urchin, and in the absence of signaling via this pathway, all cells in the embryo assume an ectodermal/neuroectodermal default pathway.

During early development of the sea urchin, nuclear accumulation of β-catenin occurs in the micromere and macromere lineages and the specific requirements for cWnt signaling in each of these cell tiers have been systematically examined [24,48]. Logan *et al*. [24] specifically addressed the importance of cWnt signaling for micromere function by transplanting nuclear β-catenin-depleted donor micromeres onto the mesomeres at the animal pole of host embryos. These experiments clearly showed that active cWnt signaling in the micromeres is necessary for their endomesoderm-inducing activity and for their ability to undergo skeletogenesis. Since inhibition of cWnt signaling led the micromeres to assume ectoderm-like fates, we asked whether ectopic activation of cWnt signaling in mesomeres could endow these cells (which normally lack appreciable inductive activity) with micromere-like properties. It is interesting that our results showed that nuclear accumulation of β-catenin is sufficient to respecify the mesomeres, allowing these cells to behave like micromeres with the ability to induce ectopic endomesoderm and to cell-autonomously give rise to cells with skeletogenic properties.

To better understand these results, it is useful to review earlier studies relevant to this work. Previous investigators observed the effects of lithium, a chemical now known to activate cWnt signaling [30] in groups of isolated mesomeres [28,29,49-52]. In classic studies using the clypeasteroid sea urchin *Echinocyamus pusillus*, Von Ubisch [28,49] found that AHs exposed to lithium formed endoderm and spicules. Depending on the concentration of lithium used, different phenotypes were obtained in the AH explants. For example, AHs exposed to low levels of lithium formed a stomodeum and a short archenteron, whereas those exposed to higher levels often formed a nearly normal pluteus. At even higher concentrations, von Ubisch found that lithium treatments caused the AHs to become vegetalized exogastrulae. More recently, Wikramanayake *et al*. [51] used molecular markers to analyze the phenotypes produced in AHs from *Lytechinus pictus* embryos to confirm that different concentrations of lithium elicited distinct patterns of gene expression and morphogenesis in these explants. One conclusion from these studies is that lithium has concentration-dependent effects on patterning the AP and DV axes in the sea urchin embryo, and since the cWnt pathway is a known target of this chemical, differential activity of this pathway could have similar effects on the early patterning of the embryo.

The above studies did not address whether lithium-treated mesomeres acquired the ability to transmit non-cell autonomous inductive signals, but this question was addressed in experiments done by Hörstadius [2,11]. In these experiments, Hörstadius examined the effects of recombining lithium-treated mesomeres with isolated AHs in the sea urchin *Paracentrotus lividus*. Hörstadius recombined lithium-treated an2 progeny (the vegetal-most tier of mesomeres in the 32-cell stage embryo) with isolated AHs of untreated embryos and found that the chimera developed into a nearly normal pluteus with a tripartite gut, spicules and a normal AP and DV axis. The lithium-treated cells contributed to the archenteron tip, but the other endodermal cells formed in response to an inductive cue from the treated cells.

Direct activation of cWnt signaling in AHs by microinjecting *actβ-cat* mRNA confirmed that different levels of nuclear β-catenin produced distinct responses in mesomeres consistent with patterning along the AP and DV axes. In these experiments cWnt signaling was activated in isolated AHs by microinjecting *actβ-cat* mRNA (0.05 to 0.1 pg) into fertilized eggs and then isolating AHs at the 8-cell stage [25]. In many cases, the isolated mesomeres expressing actβ-cat gastrulated and activated both early and late markers of endoderm formation. When lower concentrations of *actβ-cat* mRNA (0.01 to 0.02 pg) were expressed in isolated AHs, these explants polarized along the oral-aboral axis by forming a stomodeum, a ciliary band, and activating aboral ectoderm-specific gene expression in the absence of any detectable endomesodermal gene expression. These studies clearly established a concentration-dependent activation of patterning events by cWnt activation, but they did not provide any insight into cell autonomous and cell non-autonomous mechanisms activated by this pathway during early embryogenesis.

In the current study we have demonstrated that normal development up to the pluteus larval stage can be recovered by just exposing isolated AHs to a pair of mesomeres expressing nuclear β-catenin. In these chimeras, the AP and DV axes are recovered and endomesoderm is specified, resulting in the formation of what appears to be a normal pluteus within 3 days. Our experiments differ a little from those done by Hörstadius because we performed the cell transplantations at an earlier stage. In his investigation, Hörstadius recombined mesomeres exposed to lithium with the untreated AH at 32 hpf. In our experiments, *actβ-cat* mRNA-injected embryos were recombined with uninjected AHs at the 16-cell stage. In both cases, when experimentally manipulated mesomeres (either *actβ-cat* mRNA-injected or lithium-treated) were placed next to the untreated AH, the treated mesomeres acted in many ways like an organizing center. Mesomeres that expressed activated β-catenin were able to induce formation of endoderm and NSM in neighboring cells and embryos formed proper AP and DV axes. The ability to induce endomesoderm is not a property unique to the micromeres. Ectopic activation of cWnt signaling in a set of mesomeres is sufficient to endow these cells with the ability to induce endomesoderm. Our results and the transplantation studies of Hörstadius (recombining lithium-treated mesomeres with the vegetal portion of 32-cell stage AHs) support this conclusion. In both experiments, only activation of the cWnt pathway in the transplanted mesomere pair was necessary to impart endomesoderm-inducing activity to these cells and no additional signals were needed. This suggests that it is primarily activation of the cWnt pathway, and not some other molecular component specific to the micromeres that is needed to induce proper formation of the embryonic body axes.

At the molecular level, how might selective activation of canonical Wnt signaling in the mesomeres activate formation of the SM? To address these questions, it is helpful to refer to the Sea Urchin Endomesoderm Gene Regulatory Network [53], a systems level analysis of the interactions between key signaling pathways and transcription factors that regulate formation of endoderm and mesoderm during early development of the sea urchin. Several key molecules are likely to be activated in the mesomeres injected with *actβ-cat* mRNA. One micromere-specific gene activated by cWnt signaling is *Pmar1*. Although *Pmar1* is not typically expressed in the mesomeres, it is thought to be upregulated in the presence of cWnt signaling due to the fact that: A) in normal development *Pmar1* expression increases in portions of the embryo experiencing cWnt signaling [54]; B) *Pmar1* is downregulated when cWnt signaling is blocked by overexpression of the cytoplasmic tail of cadherin [53]; and C) the cis-regulatory region of the *Pmar1* gene contains a Tcf binding site [54]. Specification of the SM depends on expression of *Pmar1*. If *Pmar1* is overexpressed throughout the embryo, all of the cells, including the mesomeres, are transformed into SM [55]. Pmar1 activates expression of skeletogenic regulatory genes by inhibiting the repressor HesC (a protein distributed throughout the embryo except in areas experiencing cWnt signaling) [56]. In the absence of Pmar1, HesC represses SM formation. When HesC is inhibited by Pmar1, HesC no longer represses transcription of skeletogenic regulatory genes such as *Ets1, TBr,* and *Dri*[53,55] and this allows the micromeres to form SM. In our work, the mesomeres expressing actβ-cat likely experience upregulation of Pmar1, setting into motion the series of events needed to allow these cells to differentiate into primary mesenchyme.

In a previous study Sweet *et al*. [57] performed similar experiments to those in our studies, but in those experiments mesomeres injected with *Lytechinus variegatus Delta* mRNA were recombined with untreated AHs. Delta is the critical signal from the micromeres that segregates the veg2 endomesoderm into the more vegetal NSM layer and the more animally placed endoderm tier. Delta/Notch signaling is also later required in the endoderm for differentiation of this tissue. Interestingly, recombination of Delta-expressing mesomeres with isolated AHs resulted in normal plutei similar to the results of our experiments. However, in the experiments of Sweet *et al*. [57] the Delta-expressing cells did not contribute to the SM. The origin of the micromere-like cells in their chimeras produced by Sweet *et al.*[57] is not known, but they may be a result of the lineage conversion of NSM cells induced by Delta. The ability of a population of NSM cells to convert to an SM fate in the absence of endogenous SM cells has been well-documented in sea urchins [58].

### The cell fates induced in mesomeres differs with *β-catenin* mRNA concentration

Like von Ubisch [28,49] and Wikramanayake *et al*. [25], we found that the extent to which the cWnt pathway is activated affects cell fates induced in isolated AHs. While we have not carried out extensive analyses of the effects of different concentrations of *actβ-cat* mRNA on the signaling capacity of respecified mesomeres, we found that there seems to be an optimum concentration of *actβ-cat* mRNA for these cells to express properties similar to those seen in endogenous micromeres. When injected with 0.5 pg/pL *actβ-cat* mRNA, mesomeres gave rise to cells that could induce endomesoderm in the untreated AH, and these cells were also competent to cell-autonomously produce spicules. However, at high concentrations of *actβ-cat* mRNA (1.0 pg/pL) injected mesomeres showed a decreased ability to form a partial or complete gut, but most (59%) retained the ability to produce spicules. Although the frequency of pigment cell formation differed slightly between the two groups, both had comparable levels of mesenchyme-like cells and polarized swimming. Higher levels of cWnt signaling in the mesomeres appear to decrease the ability of these cells to induce endoderm and pigment cells.

The concentration-dependent effects of actβ-cat on mesomere-derived cell fates may reflect the differing needs for cWnt signaling during normal embryogenesis. The role for early cWnt signaling in segregating endoderm/endomesoderm from ectoderm has been documented in a number of taxa (reviewed in [17]), but more recent work in ascidians [59] and echinoderms [60,61] has also shown a clear need for differential cWnt signaling in segregating the mesoderm from the endoderm. The loss of endoderm-inducing activity in the mesenchyme-like cells derived from mesomeres injected with high levels of *actβ-cat* mRNA may reflect the properties of endogenous micromere cells as they transition from an early signaling center to their later roles in NSM segregation and skeletogenesis. These transitions may require different levels of cWnt activation. However, more studies are needed to carefully analyze these possibilities.

### Implications of localized cWnt-dependent signaling for the evolution of the metazoan body plan

Pattern formation along the AP axis in many bilaterian taxa is influenced by the maternally specified AV axis. In general, the animal pole-derived blastomeres give rise to the epidermis and the central nervous system and the vegetal pole-derived blastomeres give rise to the endomesoderm [62]. Recent work has shown that cWnt signaling in vegetal blastomeres is critical for endoderm/endomesoderm specification in echinoderms [24,25,34], ascidians [59,63,64], hemichordates [65], mollusks [66], and nemerteans [19]. Moreover, the role for cWnt signaling in specification of the posterior end of the embryo has been well established in vertebrates (reviewed in [17,23]) and regenerating planarians [67-69]; however, the roles of this pathway during embryogenesis in the planarian *Schmidtea polychroa* did not clearly indicate that it acts in early endomesoderm specification [70] and warrant further investigation. Intriguingly, in cnidarians, the closest outgroup to the Bilateria, cWnt-dependent endoderm specification occurs at the opposite side of the embryo, at the animal pole [71]. Endoderm specification also occurs at the animal pole in ctenophores, another early diverging metazoan taxon that is an outgroup to the bilaterians and cnidarians [71]. The molecular basis for endoderm specification in ctenophores is not known, but the observation that endoderm specification occurs at the animal pole in two non-bilaterian phyla has led to the proposal that endoderm segregation and gastrulation was located at the animal pole early in the evolutionary process before it became located to the vegetal pole in the bilaterian lineage [72-74]. It is likely that this involved a shift in the site of activation of cWnt signaling from the animal pole to the vegetal pole. This idea is supported by numerous studies investigating the evolution of body axis formation that have shown that the role of cWnt signaling in establishing the primary body axis is conserved and that a shift in the location of cWnt signaling during early embryogenesis occurred multiple times in the evolution of metazoans [17,73,75].

The current study is significant because it experimentally demonstrates that polarity in the sea urchin embryo, as well as specification of the site of endomesoderm formation, can be regulated by simply shifting nuclear accumulation of a single molecule, β-catenin to an ectopic site. This supports the idea that shifting the site of early cWnt signaling could have been sufficient to allow formation of the endomesoderm in a new location, and may provide an explanation for the apparent abrupt transition from pre-bilaterian to bilaterian body plans during animal evolution.

## Conclusions

In summary, this study has demonstrated that the endomesoderm-inducing ability of micromeres can be replicated in a pair of mesomeres by selectively activating the cWnt pathway in these cells. By simply shifting the distribution of β-catenin from the cytoplasm to the nucleus, we have transformed the mesomeres into an organizer-like signaling center capable of patterning a normal embryonic body axis when recombined with a field of mesomeres. Inductive effects of the actβ-cat-expressing mesomeres were concentration-dependent and higher concentrations were less likely to allow formation of the endoderm. Finally, this work is consistent with the hypothesis that a shift in the site of early cWnt signaling during early embryogenesis could modify polarity of the main body axes. It is possible that a mutation changed the location of early cWnt signaling, contributing to the evolution of the bilaterian form from the pre-bilaterian form.

## Abbreviations

Actβ-cat: Activated β-catenin; AH: Animal half; ANE: Anterior neuroectoderm; AP: Anterior-posterior; AV: Animal-vegetal; cWnt: Canonical Wnt; DV: Dorsoventral; GSK-3β: Glycogen synthase kinase-3β; hpf: Hours post fertilization; NSM: Non-skeletogenic mesoderm; PNE: Posterior neuroectoderm; RDX: Rhodamine dextran; SM: Skeletogenic mesoderm.

## Competing interests

The authors have no competing interests.

## Authors’ contributions

CAB and AHW both linearized and transcribed *actβ-cat* mRNA, microinjected, performed the microsurgeries, collected images for this paper, and participated in writing of the manuscript. Both authors have read and approved the final manuscript.
